# Hyena Bite Injury to the Neck Leading to Laryngotracheal Separation

**DOI:** 10.7759/cureus.29104

**Published:** 2022-09-13

**Authors:** Robert J Macielak, Katerina J Green, Seid Temam, Joshua P Wiedermann

**Affiliations:** 1 Otolaryngology - Head and Neck Surgery, Mayo Clinic, Rochester, USA; 2 Plastic and Reconstructive Surgery, University of Pittsburgh Medical Center, Pittsburgh, USA; 3 Otolaryngology - Head and Neck Surgery, Mekelle University, Mekelle, ETH

**Keywords:** hyena attack, hyena bite, laryngotracheal separation, laryngeal trauma, global surgery

## Abstract

Animal-induced trauma can lead to severe injury and death, especially in medically isolated settings. Few reports of hyena attacks on humans have been reported in the literature. The goal of this report is to describe such an attack and the heroic efforts required to preserve life and function in a resource-limited environment.

A 55-year-old female was attacked by a hyena in a rural region of Ethiopia. Despite delays in medical care, she was able to survive this attack and was successfully discharged after prolonged treatment efforts.

Animal-induced trauma is a potential source of substantial and disfiguring injury, especially in resource-limited environments. Early transfer to tertiary care centers and creative solutions are needed to optimize outcomes in such environments.

## Introduction

The spotted hyena (Crocuta crocuta) is a ubiquitous carnivore in Sub-Saharan Africa [1]. Despite typical preconceptions, hyenas are not solely scavengers and will elect to hunt, without reservation or distinction of prey, if so required [2]. There have been few reports of actual attacks on humans [3-5], but this likely represents an underrepresentation of the true burden of this incident given the likely location and settings where these events occur.

Prior reports examining such attacks have noted distinct features of hyena aggression [3-5]. Typically, these predators seek out vulnerable targets, e.g., children and sleeping victims, during the evening or night [3,4]. Unlike big cat predators, which typically grab and compress the neck or face, hyenas tend to grab and rip from these areas-much like domestic dog injuries but with far greater force [3]. When applied to humans, this hunting technique has led to mutilating injuries that cause disfigurement and substantial detriment to quality-of-life, if not the outright cause of death [3,4]. The following report describes a scenario in which a woman survived a hyena bite injury to the neck and associated resource-induced delays in care.

## Case presentation

The patient is a 55-year-old female from a nomadic tribe in the Afar region of eastern Ethiopia with no reported past medical history. The patient was attacked while sleeping outside, as is typical of her society. The patient was found to have a large defect of the anterior neck with associated claw marks to the chest and abdomen. At a local hospital, the patient was resuscitated with intravenous fluids and direct compression for hemostasis. For unknown reasons, no further management of her injuries or resuscitative efforts occurred. Despite this, the patient survived this initial threat to her life, and she was transported to the nearest tertiary medical facility 10 days after the attack.

**Figure 1:**
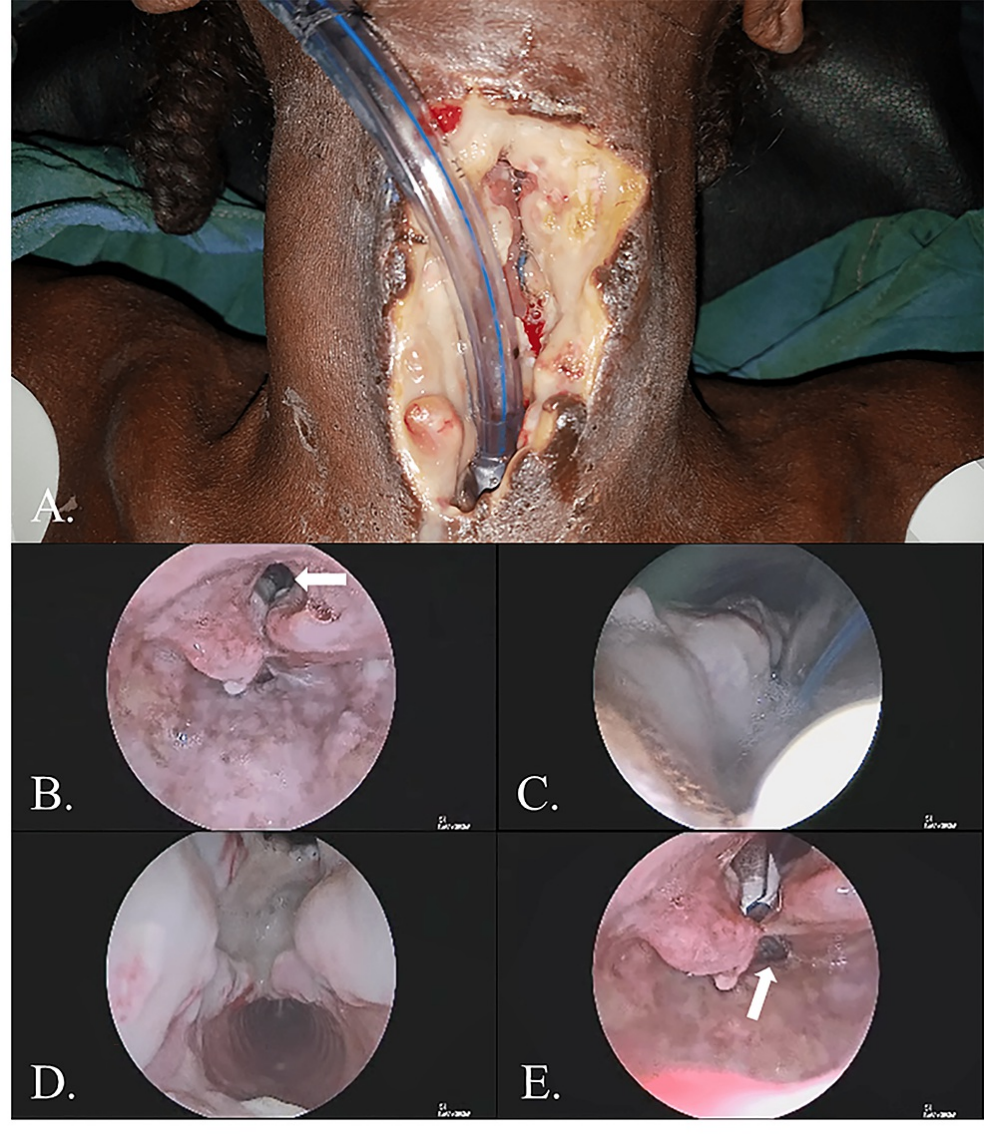
Preoperative photograph and endoscopic images showing the extent of the anterior neck injury. (A) Photograph depicting the injury and (B) endoscopic view of the patient’s supraglottis. A complete absence of the true vocal folds is evident (white arrow). (C) Endoscopic view of the patient’s proximal trachea. The anterior tracheal wall injury can be easily visualized. (D) Endoscopic view of the distal extent of the patient’s injury. Note the normal appearance of the distal airway. (E) Endoscopic view of the patient’s pharynx showing communication between the anterior neck defect and the proximal esophagus (white arrow).

Upon presentation, the patient’s anterior neck was notable for an infected wound with obvious communication with the airway. Given the patient’s condition, initial management focused on source control and securing the patient’s airway distally. Direct laryngoscopy and rigid endoscopy noted an absence of the glottis, subglottis, cricoid, and approximately 2/3 of the anterior tracheal wall for a length of six tracheal rings. There was also noted to be a violation of the anterior esophageal wall (Figure 1). A nasogastric tube was placed, and necrotic tissue within the wound bed was debrided. The patient’s anterior esophageal wall and supraglottis were then closed in a running locking fashion where possible. The patient’s distal trachea was secured to the skin, and the remnant proximal trachea and skin were closed. The superior portion of this closure was left open to allow for salivary egress. Lastly, the patient’s distal airway was cannulated with a tracheostomy tube.

**Figure 2:**
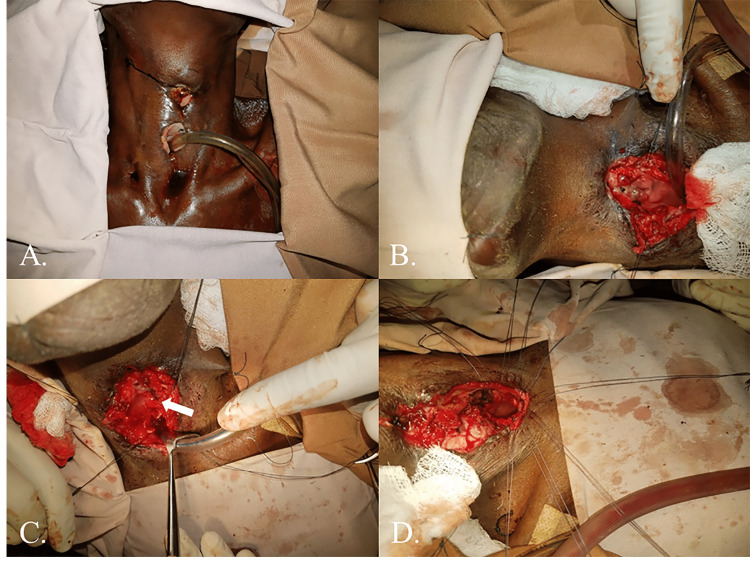
Photographs documenting the second reconstructive surgery. (A) Preoperative image showing appropriate recovery from the initial surgery. (B) Image depicting full exposure of the defect after reopening. (C) Image depicting closure of the remnant supraglottis after appropriate removal of excess tissue (white arrow). (D) Image depicting the formal maturation of the tracheostoma.

The patient subsequently underwent intensive therapy with intravenous antibiotics and electrolyte replacement. The antibiotic choice was complicated by the inability to obtain a bacterial culture given limited local resources as well as the absence of defined pathogenic bacteria after hyena attacks. Enteral feeding through the nasogastric tube consisted of whole milk, water, and multivitamins per available resources. After approximately four weeks of treatment, the patient recovered to the point that further definitive operative efforts could be entertained. Thus, formal laryngotracheal separation with the closure of persistent salivary fistulae was completed. The patient’s tracheostoma was matured, and remnant tracheal cartilage and mucosa were removed from the patient’s wound to allow for complete separation from laryngeal structures. Lastly, remnant salivary fistulae were demucosalized and over-sewn (Figure 2).

**Figure 3:**
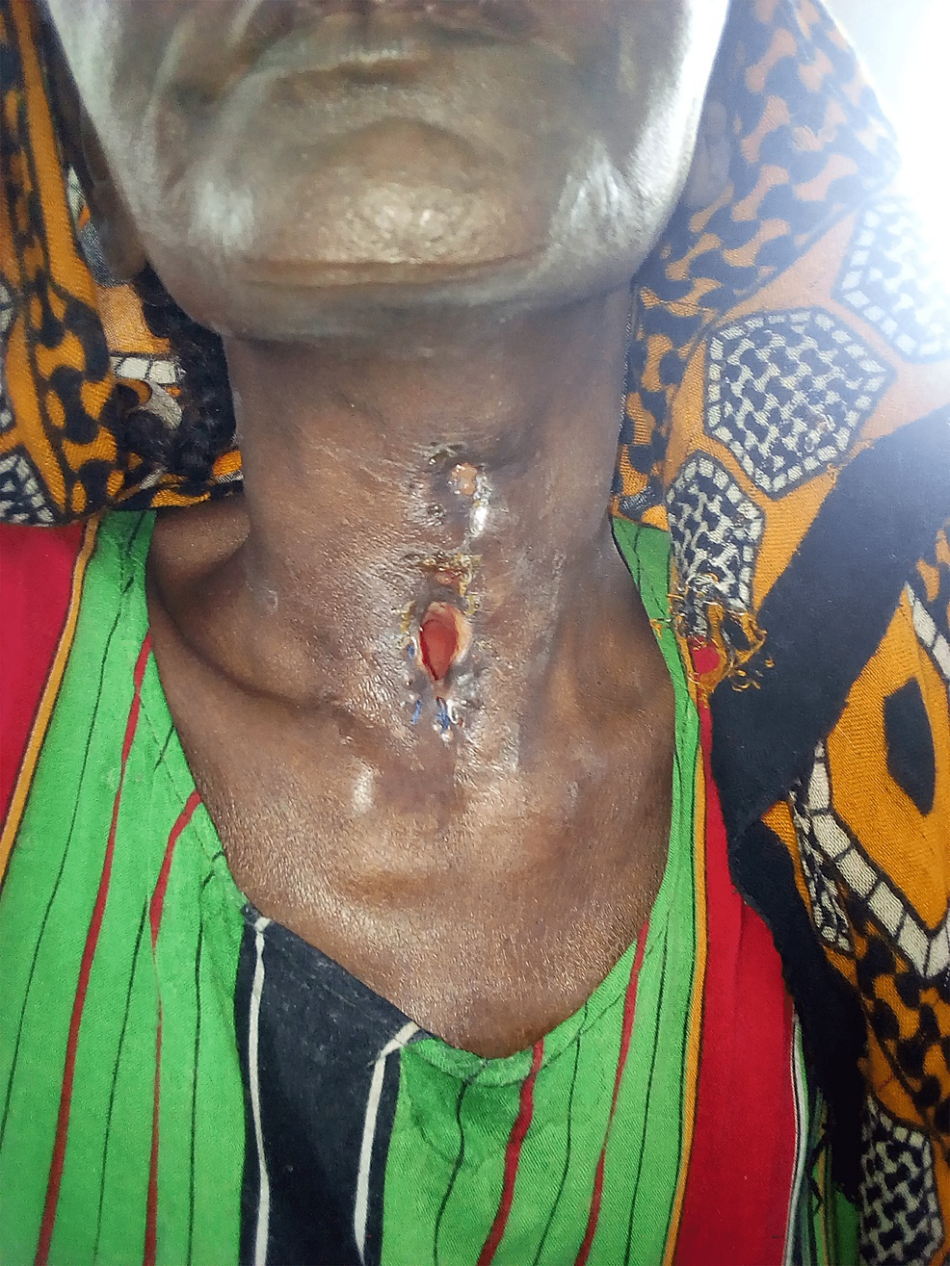
Photograph showing the patient’s wound after healing. Note that the area of prior fistula has largely healed with no continued drainage appreciated.

The patient tolerated this procedure well, and she recovered appropriately. After three weeks, a swallow study showed no apparent salivary leak, and the patient’s nasogastric tube was removed. The patient subsequently remained hospitalized to assure healing, physical rehabilitation, and adaptation to a nomadic life without a larynx. Although she was illiterate, she learned to communicate through hand-signaling and drawing pictures. Approximately three months after the initial trauma, the patient was discharged from the hospital, tolerating a full oral diet (Figure 3). She was given a metallic tracheostomy tube to maintain the patency of the stoma. The patient has unfortunately been lost to follow-up since this time.

## Discussion

The presented case is an extreme example of animal-induced trauma to the neck. The nature of the patient’s injury, with wide entrance into the airway and a lack of damage to the critical vasculature of the neck, allowed for the patient to survive the initial attack despite limited resuscitative efforts. A literature review has identified only five prior cases of reported hyena-induced trauma to the head and neck [3-5]. Notably, however, this appears to be the first reported hyena-induced trauma to the neck, and, given this paucity of literature, likely reflects one of the first observed cases of survival after such a dramatic animal-induced injury.

Situations such as this exemplify the creative solutions required to practice in isolated medical environments. Physical barriers to appropriate care combined with the known shortage of resuscitation experience and resources placed this already critically ill patient at additional risk [6,7]. Much like in acute trauma, rapid and directed stabilizing surgery was necessary to identify the extent of injury, secure the airway, and obtain source control. Postoperatively, medical optimization was performed utilizing the limited resources available-even at a tertiary center-including milk and multivitamins rather than standard tube feeding formula. Even after the successful intervention, given the patient’s nomadic culture, prolonged hospitalization was required to allow for the patient to acclimate to her new airway and to aid in future communication given her illiteracy. Considering the low likelihood of future follow-up in such a setting, this assurance of safety and ability to communicate was vital. In the end, the patient’s successful discharge was a testament not only to the collaborative resuscitative efforts put forth but also to the patient’s determination during the long recovery period.

## Conclusions

Animal-induced trauma is a potential source of substantial and disfiguring injury, especially in medically isolated locations. The burden of these injuries is likely under-reported due to their life-threatening nature. In instances of patients reaching medical care, early resuscitation followed by management at a tertiary center is key to survival when facing severe airway trauma in resource-limited environments. Our experience highlights the resilience required by both the patient and the medical team to overcome barriers of distance to tertiary care centers, resource shortages, and restrictions on follow-up.
